# In Vitro Effects of Beta-2 Agonists on Skeletal Muscle Differentiation, Hypertrophy, and Atrophy

**DOI:** 10.1097/WOX.0b013e31825eff8b

**Published:** 2012-06-15

**Authors:** Francesca Wannenes, Loretta Magni, Matteo Bonini, Ivan Dimauro, Daniela Caporossi, Costanzo Moretti, Sergio Bonini

**Affiliations:** 1 Institute of Translational Pharmacology, National Research Council, Rome, Italy; 2 Department of Medicine, "Sapienza" University, Rome, Italy; 3 Department of Health Science, University of Rome "Foro Italico", Rome, Italy; 4 Department of Internal Medicine, Endocrinology Unit, University of Tor Vergata, Rome, Italy; 5 Department of Internal Medicine, Second University of Naples, Naples, Italy

**Keywords:** beta-2 agonist, skeletal muscle hypertrophy/atrophy, asthma, COPD, physical performance

## Abstract

**Background:**

Beta-2 agonists are widely used in the treatment of asthma and chronic obstructive pulmonary disease for their effect on airway smooth muscle relaxation. They also act on skeletal muscle, although their reported ergogenic effect is controversial.

**Aim:**

To evaluate the in vitro effects of short-acting and long-acting beta-2 agonists on adrenergic receptor (ADR) expression, hypertrophy, and atrophy markers, in a skeletal muscle cell line.

**Methods:**

The C2C12 cell line was used as a model of skeletal muscle differentiation. ADR messenger RNA expression was evaluated in proliferating myoblasts, committed cells, and differentiated myotubes, in basal conditions and after treatment with 10^-6 ^M clenbuterol, salbutamol, salmeterol, and formoterol. Effect of beta-2 agonists on gene and protein expression of hypertrophy and atrophy markers was assessed in differentiated myotubes.

**Results:**

Our study shows that beta-2 ADR messenger RNA was expressed and progressively increased during cell differentiation. Beta-2 agonist treatment did not affect its expression. Skeletal muscle hypertrophy markers (fast and slow myosin, myogenin) were not modulated by any of the beta-2 agonists evaluated. However, clenbuterol induced a significant, dose-dependent downregulation of skeletal muscle atrophy genes (atrogin-1, MuRF-1, and cathepsin L).

**Conclusions:**

The reported ergogenic effect of beta-2 agonists, if any, should be considered as drug-specific and not class-specific and that of clenbuterol is mediated by the inhibition of the atrophic pathway.

## 

Because of their potent bronchodilator effect, both short-acting and long-acting beta-2 agonists (SABA and LABA) are recommended by the international guidelines [1] and widely used in the current treatment of asthma and chronic obstructive pulmonary disease (COPD) [2].

Beta-2 agonists exert their pharmacologic effects through specific G protein-coupled receptors (adrenergic receptors, ADRs), classified in beta-1, beta-2, and beta-3 (ADRB1, ADRB2, and ADRB3) preferentially expressed in cardiac tissue, airway smooth muscle and lipocytes, respectively [3].

In the airways, ADRB2 activation is followed by a specific signal transduction pathway, which ultimately results in an increase of cyclic adenosine monophosphate required for airway smooth muscle relaxation and bronchodilation [3].

ADRB2 are also present in human skeletal muscle, even though their expression during muscle differentiation has not yet been completely elucidated [4]. Human skeletal muscle undergoes a continuous remodeling process, which results from a delicate balance between synthesis and breakdown of skeletal muscle fibers [5]. This varies during growth, aging, and health status, as shown by the expression of several genes and related proteins, which are used as markers of muscle differentiation, hypertrophy, and atrophy [6,7].

It has been shown that beta-2 agonists may have a profound effect on skeletal muscle turnover, both in vitro and in vivo. Hinkle et al [8] demonstrated that clenbuterol causes hypertrophy and has antiatrophic effects in mice skeletal muscle. Similarly, Delday and Maltin [9] have shown that clenbuterol increases the expression of myogenin in immobilized rat muscles. Further studies indicated that clenbuterol also induces a shift from slow to fast myosin fibers in skeletal muscle [10].

In humans, the administration of salbutamol and salmeterol has been reported to have ergogenic effects [11-13]. These would be beneficial in patients with COPD, in whom muscle atrophy and weakness are reported [14,15]. On the other hand, although at present, there is no evidence of a significant positive effect on physical performance, [16] beta-2 agonists are included in the World Anti-Doping Agency list of banned substances and prohibited in asthmatic athletes, both in and out of competition [17]. An exception is represented for salbutamol and salmeterol as inhalers, which can be used only after a declaration of use in the presence of a documented diagnosis of asthma [17]. Despite the clinical relevance of the effects of beta-2 adrenergic drugs on skeletal muscle, in vitro and in vivo studies in this area are limited. Furthermore, the effects of a single beta-2 agent are often extrapolated to other beta-2 agonists and to different dosages and administration routes.

We report here the results of a study aimed at evaluating the in vitro effects of the 2 major agonists, short-acting beta-2 agonist (SABA) (clenbuterol and salbutamol) and long-acting beta-2 agonist (LABA) (formoterol and salmeterol), on the expression of ADRB2 in a muscle cell line, as well as of some relevant markers of muscle hypertrophy and atrophy.

As a muscle cell line, we used the C2C12 that we recently validated as a model of in vitro skeletal muscle differentiation by the analysis of muscle-specific differentiation-dependent markers [18].

## Methods

### Cell Culture and Beta-2 Agonist Treatment

C2C12 mouse myoblasts were grown as described [18] and plated at a density of 2.5 × 10^4 ^cells per milliliter in growing medium (GM); myotube differentiation was induced after 72 hours by switching 80%-90% confluent cells in differentiation medium (DM). Cells were harvested for molecular analysis at 24 and 48 hours (proliferating myoblasts) and at 72 hours (D0, committed myoblasts) after seeding in GM and at 2 and 4 days (D2 and D4) after switch in DM.

To study the effects of a beta-2 agonist treatment, salbutamol, salmeterol, clenbuterol, and formoterol (Sigma-Aldrich, Milan, Italy) were diluted in GM or DM and added to the cell culture at the start of experiments and at D0. In preliminary experiments using different concentrations of the beta-2 agonists (10^-4 ^to 10^-8 ^M), 10^-6 ^M was shown to be the optimal concentration to be used in further experiments.

### RNA Extraction and Quantitative Reverse Transcription Polymerase Chain Reaction

Total RNA was extracted and reverse-transcribed as described [18]. Quantitative polymerase chain reaction (qPCR) was performed in ABI PRISM 7000 light cycler (Applied Byosistems, Monza, Italy) using SYBR Greener qPCR SuperMix for ABI Prism (Invitrogen, Milan, Italy) as indicated by the manufacturer. All primers were optimized for real-time reverse transcription PCR amplification checking the generation of a single amplicon in a melting curve assay and the efficiency in a standard curve amplification (> 98% for each couple of primers). Quantitative reverse transcription PCR sample value was normalized for the expression of 18s messenger RNA (mRNA). The relative level for each gene was calculated using the 2^-ΔΔCt ^method [19] and reported as arbitrary units. In all experiments, each sample was analyzed in triplicate. Sequences of primers are given in Table 1.

**Table 1:** Sequences of Primer Pairs Used for qPCR Measurements

Name	GENBANK	Sense Primer	Antisense Primer
18s	X00686	CCCTGCCCTTTGTACACACC	CGATCCGAGGGCCTCACTA
ADRB1	NM007419	GTAACGTGCTGGTGATCGTG	AAGTCCAGAGCTCGCAGAAG
ADRB2	NM007420	GAGCACAAAGCCCTCAAGAC	GTTGACGTAGCCCAACCAGT
Atrogin	NM026346	GCAAACACTGCCACATTCTCTC	CTTGAGGGGAAAGTGAGACG
Cathepsin L	NM009984	GTGGACTGTTCTCACGCTCAAG	TCCGTCCTTCGCTTCATAGG
Murf 1	NM001039048	ACCTGCTGGTGGAAAACATC	CTTCGTGTTCCTTGCACATC
Myogenin	NM0311891	AGCTGTATGAGACATCCCCC	TTCTTGAGCCTGCGCTTCTC
MyHC-IIb	BC052786	GAGCAGCTGGCGCTGAAGGG	GATTTCTCCTGTCACCTCTC
MyHC IIx/d	XM354615	TCAATGAGCTGACTGCGCAG	CAAGCTGCCTCTTCAGCTCC
MyHC-I	NM080728	AAGATCGTGTCCCGAGAGGG	TTGTACAGCACAGCCGGCTC

### Protein Extraction and Western Blot Analysis

Total protein was extracted as described [18]. Total extracts were separated in a sodium dodecyl sulfate polyacrylamide gel and transferred to a nitrocellulose membrane. Transfer was verified by Ponceau S staining. The membrane was blocked 60 minutes at room temperature with 1% or 5% nonfat dry milk in Tris buffered saline (T-TBS; 50 mM Tris base, 0.9% NaCl, Tween 20 0.001%; pH 7.4). Anti-skeletal myosin--fast and slow--(M4276 and M8421; Sigma-Aldrich), anti-myogenin (M3559; Dako, Milan, Italy), anti-α-tubulin (T5168; Sigma-Aldrich) antibodies were added and incubated overnight at 4°C. After a 30-minute incubation at room temperature, the membrane was washed 3 times with T-TBS and incubated for 60 minutes at room temperature with horseradish peroxidase (HRP) labeled anti-rat or anti-mouse (Jackson Immunoresearch, LiStar-Fish, Milan, Italy) in 5% nonfat dry milk. The membrane was then washed 3 times, and the antibodies were visualized using ECL Western Blotting Detection Reagents (Amersham Biosciences, Milan, Italy). Quantitative analysis was performed using Quantity One analysis software (Versadoc 1000; Biorad, Milan, Italy); sample value was normalized for housekeeping protein (α-tubulin) and is reported as arbitrary units.

### Statistical Analysis

Data are presented as the mean ± SD of 3 different independent cell cultures in each experimental setting. Statistical analysis was performed for all the aimed outcomes, using a 2-way factorial analysis of variance and a 1-way analysis of variance followed by a Bonferroni test. *P *values < 0.05 were considered statistically significant.

## Results

### Beta-Adrenergic Receptor Expression During C2C12 Differentiation

We investigated the ADR mRNA expression and modulation in proliferating myoblasts (24 and 48 hours), committed cells (D0), and mature myotubes (D2 and D4). Both ADRB2 mRNA and, to a significantly lesser extent, ADRB1 (*P *< 0.001) mRNA were expressed by the C2C12 cell line at any time point, whereas no expression of ADRB3 was detected in our experimental conditions.

**Figure 1:**
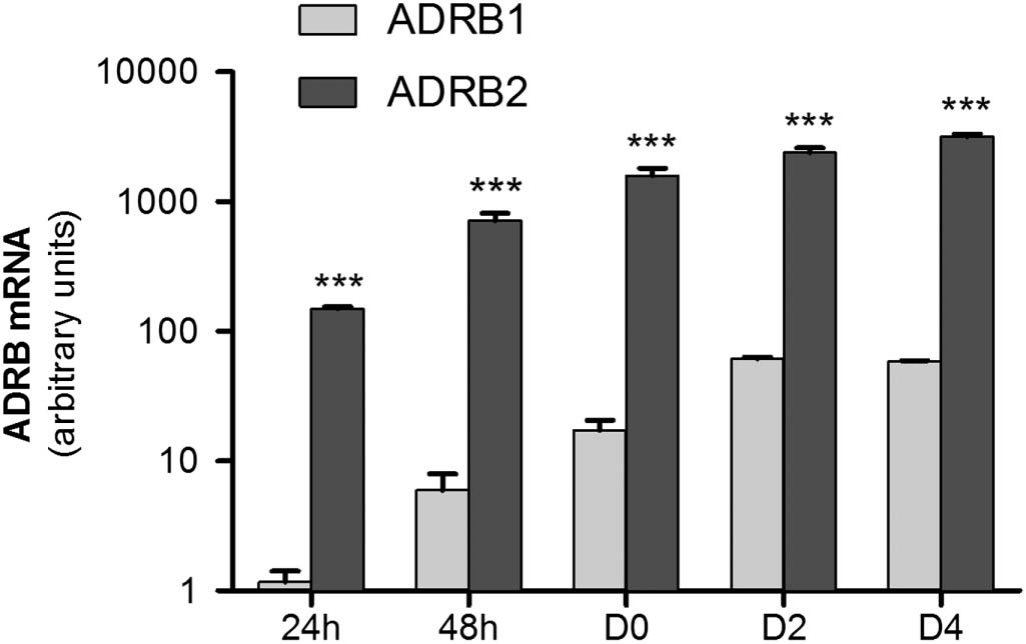
**ADRBs expression during cells differentiation**. Time course of ADRB1 and ADRB2 mRNA expression during cell differentiation analyzed by qPCR. Cells were harvested for molecular analysis at 24 and 48 hours (proliferating myoblasts) and at 72 hours (D0, committed myoblasts) after seeding in GM and at 2 and 4 days (D2 and D4) after switch in DM. The results shown represent the mean of 3 independent experiments using independent cell cultures in each experimental setting. Bars symbolize qPCR data normalized versus ADRB1 mRNA expression value in untreated cells. ADRB2 were significantly more expressed than ADRB1 (*P *< 0.001). The significant increase of ADRB2 expression (*P *< 0.001) refers to the previous time point of culture (data normalized versus the baseline values). ***P < 0.001.

qPCR data normalized versus mRNA ADR basal expression showed that ADRB2 mRNA progressively and significantly (*P *< 0.001) increased during the differentiation process of the cell culture (5-folds from 24 to 48 hours, 2 folds from 48 hours to D0, and 1.5-fold from D0 to D2), remaining high in terminally differentiated myotubes (Figure 1).

### Effect of Beta-2 Agonist Treatment on ADRB2 Expression

**Figure 2:**
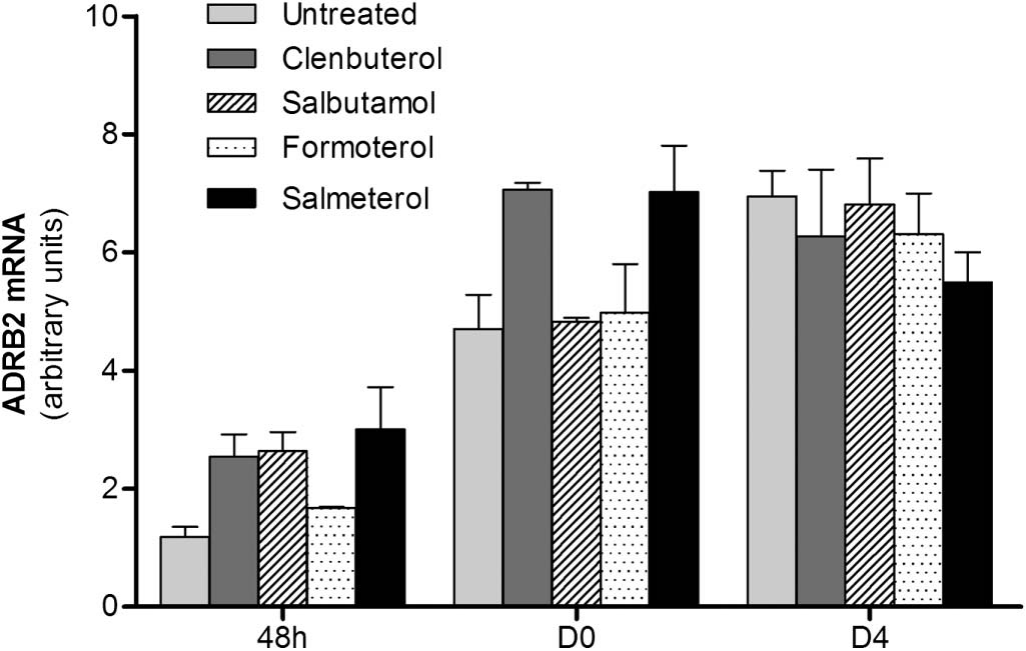
**Beta-2 agonists effects on ADRB mRNA expression**. Time course of ADRB2 mRNA expression by qPCR in C2C12 cells grown in presence of 10^-6 ^M beta-2 agonists treatment (at 48 hours, D0, and D4). Bars represent mean ± SD of 3 different independent experiments.

To determine if beta-2 agonist treatment could induce some ADRB2 modulation during the skeletal muscle differentiation process, we grew C2C12 cells with a 10^-6 ^M concentration of different short-lasting and long-lasting beta-2 agonists (salbutamol, clenbuterol, salmeterol, and formoterol). The addition of 10^-6 ^M concentration of beta-2 agonists did not significantly change ADRB2 mRNA expression throughout the whole differentiation process (Figure 2).

### Effect of Beta-2 Agonist Treatment on Skeletal Muscle Hypertrophy

We treated C2C12 cells with equimolar concentrations of salbutamol, clenbuterol, salmeterol, and formoterol, to evaluate whether they could modulate the expression of hypertrophic (myogenin and myosin heavy chain isoforms, MyHCs) and atrophic genes (atrogin-1, MuRF1, and cathepsin L).

#### Myogenin Expression

**Figure 3:**
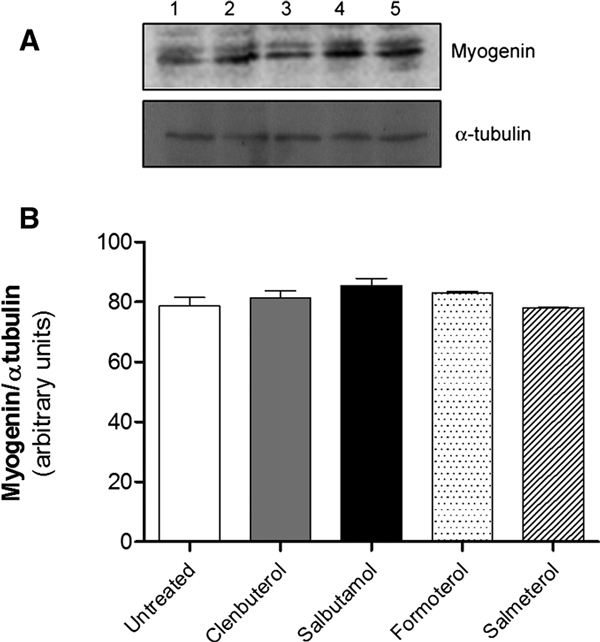
**Beta-2 agonists effects on myogenin mRNA and protein expression**. Western blot analysis of myogenin protein expression after 10^-6 ^M beta-2 agonists treatment. A, Upper panel: myogenin; lower panel: α-tubulin (loading control). Lanes: 1, untreated cells; 2, clenbuterol-treated cells; 3, formoterol-treated cells; 4, salbutamol-treated cells; 5, salmeterol-treated cells. B, Graph of quantified data from Western Blot experiments; bars show the mean ± SD of 3 different independent experiments.

Beta-2 agonist exposure did not determine a significant modulation of myogenin mRNA expression (data not shown). This lack of effect was also confirmed at protein level (Figure 3).

#### Myosin Heavy Chains Expression

**Figure 4:**
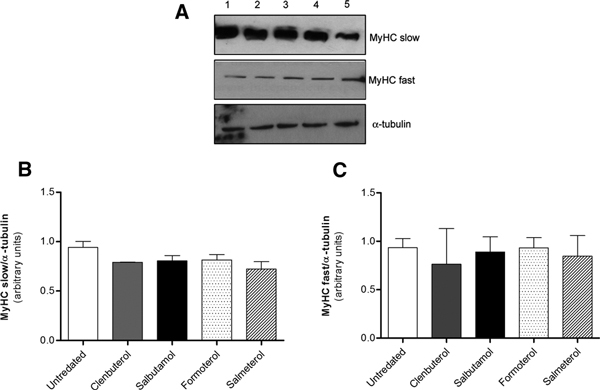
**Beta-2 agonists effects on myosin heavy chains (MyHCs) protein expression**. A, Western blotting analysis of MyHCs protein expression in differentiated myotubes. Upper panel is a representative immunoblot showing MyHC slow isoform expression; middle panel is a representative immunoblot showing MyHC fast isoform; α-tubulin (lower panel) was used as loading control. Lanes: 1, untreated cells; 2, clenbuterol-treated cells; 3, formoterol-treated cells; 4, salbutamol-treated cells; 5, salmeterol-treated cells. B, C, Graph of quantified data from Western Blot experiments with MyHC slow (B) and MyHC fast (C) isoforms. Histograms show the mean ± SD of 3 different Western blot quantifications.

qPCR analysis revealed that during differentiation, salbutamol, clenbuterol, salmeterol, and formoterol did not affect MyHCs mRNA expression (data not shown). Western blot analysis confirmed data at protein level (Figure 4).

#### Atrophic Genes Expression

**Figure 5:**
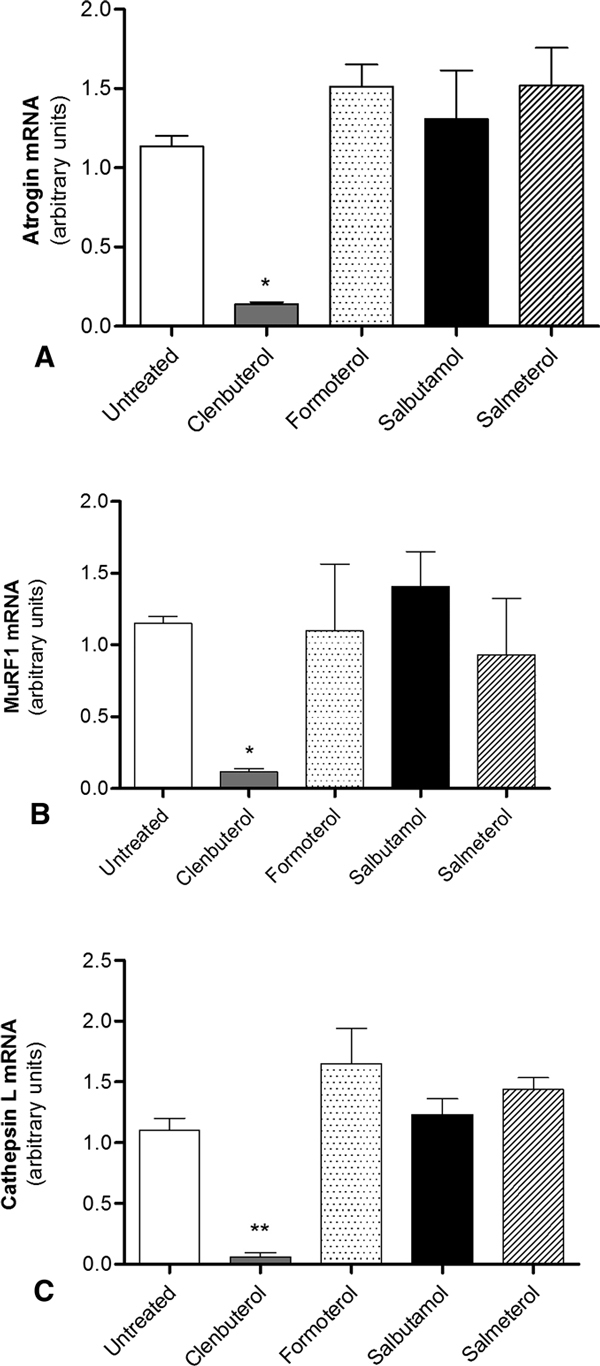
**Beta-2 agonists effects on atrophic genes mRNA expression**. qPCR analysis of atrogin-1 (A), MuRF1 (B), and cathepsin L (C) mRNA expression in terminally differentiated C2C12 cells grown in presence of 10^-6 ^M beta-2 agonists or in presence of vehicle alone (untreated). Bars represent mean ± SD of 3 different independent experiments; statistical significance was analyzed between beta-2 agonists treated versus untreated cells. *P < 0,01; **P < 0.001.

**Figure 6:**
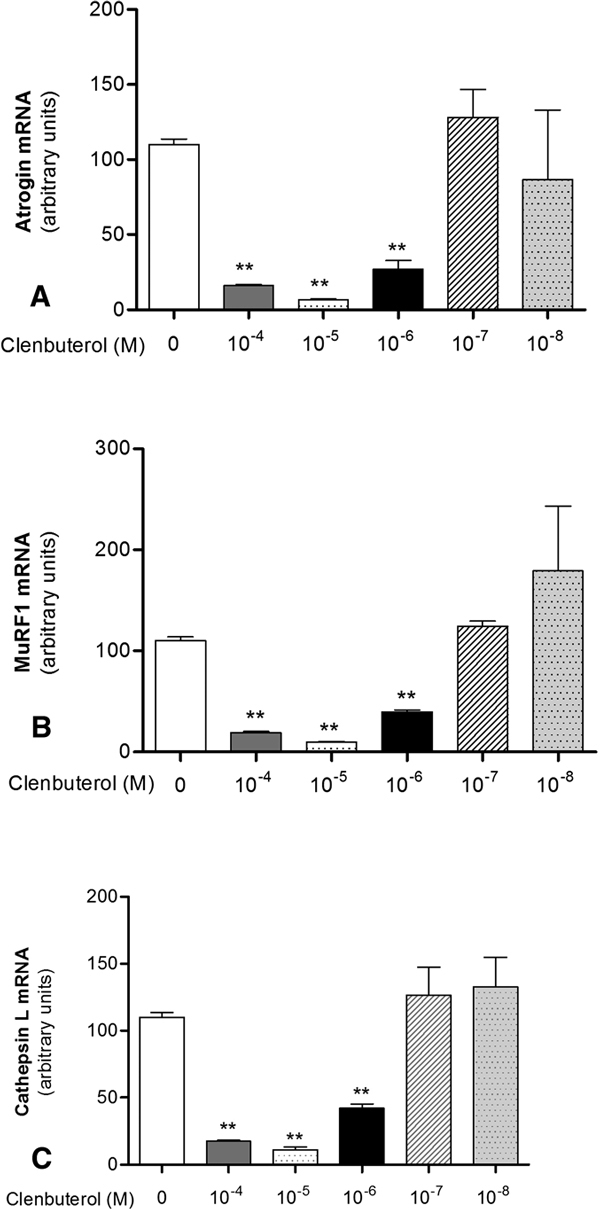
**Dose-dependent effect of clenbuterol on mRNA expression of atrophic genes**. Atrophic genes mRNA expression of atrogin-1 (A), MuRF1 (B), and cathepsin L (C) by qPCR in terminally differentiated myotubes grown in presence of 10^-4 ^M, 10^-5 ^M, 10^-6 ^M, 10^-7 ^M, or 10^-8 ^M clenbuterol or in presence of vehicle alone. Bars represent mean ± SD of 3 different independent experiments; statistical significance was analyzed between beta-2 agonists treated versus untreated cells. **P < 0.001.

qPCR analysis revealed that clenbuterol significantly decreased atrogin-1, MuRF1, and cathepsin L mRNA expression (8-, 7.5-, and 9-folds, respectively) (Figures 5A-C) in a clear dose-dependent manner (Figures 6A-C). On the contrary, salbutamol, salmeterol, and formoterol did not induce any effect on the expression of the above skeletal muscle atrophy markers (Figures 5A-C).

## Discussion

To our knowledge, this is the first study on the expression and beta-2 agonist modulation of ADRB2 in a skeletal muscle cell line in relation to subsequent differentiation stages, as well as on the comparative effects of different SABA and LABA at equimolar concentrations on hypertrophy and atrophy genes.

Our data show that transcript encoding ADRB2 are expressed in C2C12 cells in a differentiation-dependent manner, concomitant with the acquisition of a mature phenotype. Therefore, this muscle cell line may represent a useful model for studying the in vitro effects of different beta-2 agonists on skeletal muscle and specific receptor expression, as previously shown for different agonists [18]. To study the in vitro effects of clenbuterol, salbutamol, formoterol, and salmeterol, we used several genes and proteins as markers of skeletal muscle differentiation, hypertrophy, and atrophy.

Myogenin is a transcriptional factor that strictly marks terminal differentiated myotubes [20]. The myosin heavy chains (MyHCs) are terminally differentiated muscle cells markers. In normal adult skeletal muscle fibers, 4 MyHC isoforms may be expressed: 1 slow (MyHC-1) and 3 fast (MyHC-IIa, MyHC-IIb, and MyHC-IIx/d) [21]. We had previously shown that C2C12 expressed MyHC-IIb, MyHC-IIx/d, and MyHC-I mRNAs but not MyHC-IIa [18].

Transcriptional upregulation or downregulation atrophy-related genes are a characteristic feature of muscle atrophy; the most relevant atrogenes are 2 ubiquitin ligases named atrogin-1/Mafbx and MuRF1 [6]. Another important intracellular degradation mechanism in skeletal muscle is autophagy [7]. Cathepsin L is a lysosomal enzyme whose role appears to be critical for the degradation of membrane proteins and that is known to be upregulated during skeletal muscle atrophy [7].

All beta-2 agonists under investigation were shown to have no effect on ADRB2 expression and on hypertrophy genes and proteins studied. The apparent inconsistency of our results with those of previous studies [8,9] may be possibly due to different experimental systems, conditions, and concentrations of beta-2 agonists used. The 10^-6 ^M concentration we used seemed to be optimal for ADRB2 receptor expression and be related to in vivo drug concentrations of beta-2 agonists administered for therapeutic purposes.

Clenbuterol showed a dose-dependent downregulation of the atrophy markers tested. Therefore, the reported ergogenic effect of clenbuterol [8-10] is more likely due to the inhibition of the atrophic pathway rather than an effect of the drug on skeletal muscle hypertrophy.

The antiatrophic effect of clenbuterol was not shown by salbutamol, formoterol, and salmeterol when used at the same molar concentration. This finding indicates that the reported ergogenic effect of beta-2 agonists is not class specific but drug specific or related to the drug concentration used.

It is always difficult to extrapolate clinical consequences from in vitro studies to humans. However, our findings may only allow some speculations in relation to the potential benefits of using beta-2 agonists for treating muscular weakness in patients with COPD or to deny them in asthmatic athletes because of a potential ergogenic effect.

In COPD, exertional dyspnea and limited exercise tolerance represent major symptoms of the disease. Although these are largely due to the impaired lung function, wasting and dysfunction of both respiratory and peripheral muscles have long been recognized as hallmarks of the disease and important cofactors in influencing its morbidity and mortality, independently from the decline of lung functions [15,22].

Understanding the mechanisms of muscle impairment is crucial to prospect adequate therapeutic intervention. Among the several factors that contribute to muscle wasting and dysfunction in patients with COPD, [15] special attention has recently been focused on systemic inflammation and its effects on protein synthesis and degradation, [23] although physical inactivity is likely to be a more relevant factor in causing muscle weakness and possibly atrophy [24]. However, the potential favorable effect of beta-adrenergic agents on muscle dysfunction in patients with COPD does not find supporting evidence neither from our data nor from interventional studies. Certainly, more adequate therapeutic approaches should be used to treat muscle wasting in COPD [25].

The reported ergogenic effect and the increasing use of beta-2 adrenergic drugs in athletes [26] induced the International Olympic Committee in 2002 to deny the use of beta-2 adrenergic agents without a documented diagnosis of asthma based on very strict criteria [27]. However, at present, only salbutamol and salmeterol are permitted as inhalers while all beta-2 agonists (including both optical isomers where relevant) are prohibited; salbutamol (maximum 1600 μm over 24 hours), formoterol (maximum 36 μm over 24 hours), and salmeterol are permitted when taken by inhalation in accordance with the manufacturers' recommended therapeutic regimen [17]. This restriction might prevent adequate the new rule answers to the concern, previously expressed by our group and other authors, that the strict World Anti-Doping Agency regulations could exclude from treatment, according to the Global Initiative for Asthma (GINA) guidelines, in athletes with asthma or exercise-induced bronchoconstriction but actual negative challenge tests to prove it [28]. In fact, there is no evidence of a positive effect of beta-adrenergic agonists on physical performance [16]. Moreover, our data do not support any ergogenic effect except for clenbuterol. Accordingly, restriction of beta2 adrenergic agents in asthmatic athletes should rather be based on their potential effects on tolerance [29] and cardiovascular side effects (particularly for LABA), [30,31] rather on the fear of a class-specific effect on the skeletal muscle.

In conclusion, our data indicate that the C2C12 cell line may represent a useful model to study the in vitro effects of beta-2 adrenergic agents on differentiation, hypertrophy, and atrophy of the skeletal muscle. In this model, clenbuterol, salbutamol, formoterol, and salmeterol do not exert any effect either on the expression of ADRB2 receptors or on the genes and proteins tested as markers of hypertrophy. Only clenbuterol shows a significant dose-dependent inhibitory effect on the atrophic pathway, which might be responsible for the ergogenic effect reported for this molecule.

## End Note

Supported by grants of the Italian Ministry of University and Research (Anti-Doping Commission and PRIN).

Preliminary data of this study were presented as an abstract at the 2009 World Allergy Congress, December 8, 2009, Buenos Aires.
